# Optimizing Electrodiagnostic Studies: The Role of Clinical History in Improving Diagnostic Concordance—A Retrospective Study

**DOI:** 10.3390/diagnostics15040436

**Published:** 2025-02-11

**Authors:** Carlos Omar López-López, Mónica Touron De Alba, María de la Luz Montes-Castillo, Janitzia Vázquez-Mellado

**Affiliations:** 1 Applied Research and Technology Institute (InIAT), Universidad Iberoamericana—Ciudad de México, Mexico City 01219, Mexico; 2 Rehabilitation Department, Hospital General de México “Dr. Eduardo Liceaga”, Mexico City 06720, Mexico; monicatouron@hotmail.com (M.T.D.A.); luzmontesc@hotmail.com (M.d.l.L.M.-C.); 3 Rheumatology Department, Hospital General de México “Dr. Eduardo Liceaga”, Mexico City 06720, Mexico; jvazquezmellado@gmail.com

**Keywords:** concordance, clinical diagnosis, electrophysiological diagnosis, electrodiagnostic studies, electromyography, nerve conduction studies

## Abstract

**Background:** Electrodiagnostic (EDx) studies are crucial for diagnosing neuromuscular diseases and are considered a complement to neurological examinations. Their effectiveness is highly dependent on the quality of the initial clinical diagnosis, medical history, and physical examination. This study aims to evaluate the concordance between clinical diagnoses and EDx findings, and to identify the primary specialties and clinical data justifying EDx referrals. **Methods:** This retrospective study included data from referral forms and EDx reports for nerve conduction studies and electromyography at Hospital General de México “Dr. Eduardo Liceaga” from January 2022 to January 2023. Statistical analyses involved descriptive statistics, Cohen’s Kappa coefficient, and binary logistic regression to assess diagnostic concordance and the influence of clinical symptoms. **Results:** Out of 1065 referral requests, 61.8% had their clinical diagnosis confirmed through EDx studies. Most requests originated from orthopedics (25.7%), rheumatology (17.5%), and neurology (17.4%). Although 79.5% of requests included symptoms, only 32.1% included a physical examination. Common diagnoses included radiculopathy (34.6%), polyneuropathy (22.7%), and carpal tunnel syndrome (22.4%). Concordance between clinical and electrophysiological diagnoses had a coefficient of 0.578 (95% CI: 0.537–0.619; *p* < 0.001). Pain and weakness were significantly associated with diagnostic confirmation (pain: OR 3.1; 95% CI: 1.79–5.48; *p* < 0.001; weakness: OR 2.46; 95% CI: 1.35–4.48; *p* = 0.003). Neurology had the highest concordance (0.853; 95% CI: 0.829–0.878), while neurosurgery had the lowest (0.502; 95% CI: 0.378–0.627). **Conclusions:** EDx studies are valuable, yet their concordance with clinical diagnoses is limited. The experience of the requesting physician significantly impacts diagnostic accuracy. Enhancing referral information is essential for improving EDx study effectiveness and reducing unnecessary procedures, thereby improving patient outcomes and managing costs.

## 1. Introduction

Electrodiagnostic (EDx) studies encompass nerve conduction studies (NCS) in various modalities (sensory, motor, late responses, repetitive stimulation) and needle electromyography (EMG). These techniques can be performed together or separately to assess nerve–muscle integrity. NCS involves placing surface electrodes over a sensory nerve territory (to record sensory nerve action potentials) or a muscle belly (to record compound muscle action potentials) after electrical stimulation of a sensory, motor, or mixed nerve. EMG, on the other hand, requires inserting a needle electrode into a muscle belly to evaluate spontaneous and voluntary muscle activity, analyzing the characteristics of the motor unit action potential [1].

EDx studies play a critical role in diagnosing neuromuscular disorders by determining the lesion’s pathophysiology (demyelinating or axonal), identifying affected fibers (sensory, motor, or mixed), and assessing lesion distribution (mononeuropathy, multiple mononeuropathy, polyneuropathy, neuromuscular junction disorders, anterior horn cell diseases, etc.) [2,3]. Despite their significance, EDx studies do not replace a thorough medical history or neurological examination; rather, they complement them [4]. Proper use of EDx studies enhances clinical correlation, improves diagnostic accuracy, and serves as a powerful diagnostic tool [5].

Referral for EDx studies is warranted when concerning symptoms arise, such as weakness, acute onset, asymmetric presentation, or rapid progression. Additionally, EDx is indicated when symptoms persist or progress despite treatment, even if initial diagnostic tests yield negative results. A normal EDx result reduces the likelihood of peripheral neuropathy, although these studies are more sensitive in detecting conditions affecting large, myelinated fibers [6].

Despite their usefulness, EDx studies present several limitations. They require specialized equipment, highly trained personnel, and expertise in neurophysiology. Furthermore, these studies are operator-dependent, costly, and require a complex interpretation process. Therefore, EDx should be conducted by physicians specialized in neuromuscular disorders who possess in-depth knowledge of anatomy, physiology, electrophysiological procedures, and data interpretation [7,8].

The accuracy of EDx studies is directly linked to the quality of the initial clinical evaluation. A well-structured medical history and neurological examination guide the strategic planning and execution of these tests, ultimately enhancing their diagnostic yield [9]. Indeed, supplementary clinical history obtained through interviews and physical examinations provides crucial insights that refine the diagnostic approach [10].

However, in clinical practice, physicians frequently encounter patients with known symptoms but uncertain diagnoses. Requests for EDx studies based solely on symptoms—without a well-established clinical correlation—significantly reduce their diagnostic accuracy. Moreover, this practice increases the demand for EDx studies with poorly formulated requests and insufficient clinical data, ultimately leading to inefficient use of healthcare resources [11].

The aim of this study was to evaluate the concordance between clinical and electrophysiological diagnoses, as well as to identify the main medical specialties and the clinical data that justified the referral of the patient to the electrodiagnostic service.

## 2. Materials and Methods

This was a retrospective and transversal study that included the records of the electrodiagnostic laboratory of the Hospital General de México “Dr. Eduardo Liceaga” (HGMEL) from January 2022 to January 2023. This hospital is part of the public healthcare system, which offers partial coverage to the general population. Patients were referred from other departments within the hospital, including rheumatology, neurology, orthopedics, pain clinic, rehabilitation, and others.

For this study, only requests for nerve conduction and electromyography studies were selected, excluding other electrophysiological studies including evoked potentials (somatosensory, visual or auditory), repetitive stimulation test, blink reflex, and sympathetic skin response.

**Figure 1:**
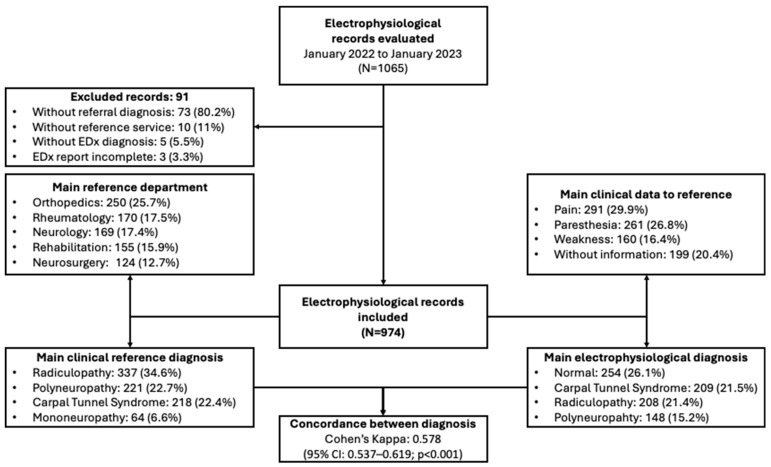
Flowchart of evaluated and analyzed electrophysiological reports.

Requests received at the laboratory should contain the following information: a clinical reference diagnosis, a signed informed consent form from the patient, identification data (name, age, sex, file number), the referring service, and the reason for the study. Requests that were illegible and did not allow for proper identification of the information, those for pediatric patients, and those with an inconclusive or incomplete electrophysiological diagnosis were excluded (Figure 1).

Unfortunately, the present study relies exclusively on requests received at the electrodiagnostic laboratory, which limits access to detailed sociodemographic data (including race, educational level, socioeconomic status, etc.).

The electrophysiological tests were performed by a team of specialized physicians, all of whom were physiatrists from the Department of Physical Medicine and Rehabilitation and certified experts in EDx medicine. In this laboratory, conduction studies include the recording of compound muscle action potentials, sensory nerve action potentials, and late responses (F-wave and H-reflex). Additionally, for electromyography studies, a nerve conduction study is conducted prior to needle electrode examination, the number of muscles examined depends on the clinical context and the findings obtained during the study. All studies strictly follow the technical recommendations of the American Association of Neuromuscular and Electrodiagnostic Medicine [12]. The scope of the EDx study is determined by the presumptive diagnosis or the physical examination conducted in the laboratory.

We analyzed the frequency of the different clinical and EDX diagnoses and compared the EDX test outcomes based on the referral diagnoses and the specialty of the referring doctor. Additionally, clinical data were analyzed with a focus on symptoms, physical examination findings, and the main reason for referral. This project was approved by the Institutional Review Board at our hospital (ID project: DECS/JPO-CT-1672-2023).

Statistical Analysis: Demographic and clinical variables were summarized using descriptive statistics, including means ± standard deviation (SD), median and interquartile range (IQR) for continuous variables, and proportions for categorical variables. Concordance was evaluated using Cohen’s Kappa coefficient and its IQR. Finally, binary logistic regression was performed to determine the association of the main signs and symptoms with the confirmation or rejection of the clinical diagnosis. A significance level of 0.05 (two-sided) was considered, and statistical analysis was conducted using SPSS for MAC version 20.

## 3. Results

A total of 1065 referral requests were included, with the majority originating from the orthopedic service (*n* = 250; 25.7%), followed by rheumatology (*n* = 170; 17.5%), neurology (*n* = 169; 17.4%), and rehabilitation (*n* = 155; 15.9%). Most of these requests involved women patients (*n* = 668; 68.6%) with a mean age of 50.1 (13.6) years old.

**Figure 2:**
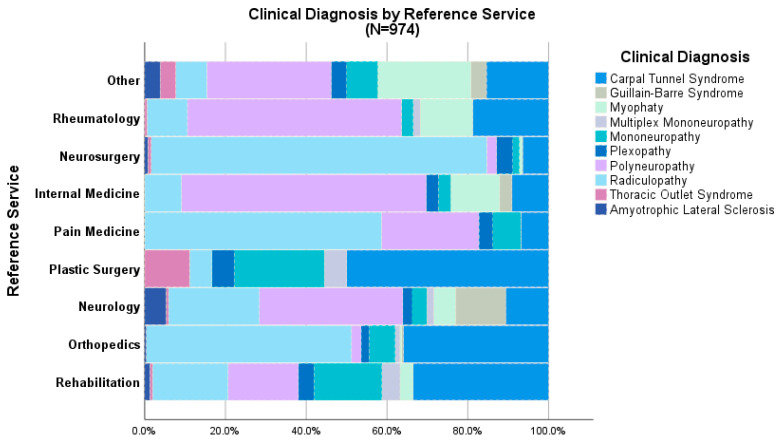
Clinical diagnosis according to Reference Service.

The main requested study was nerve conduction velocities (388, 39.8%), followed by electromyography (353, 36.2%) and electroneuromyography (233, 24%). The most frequent referral diagnoses were (Figure 2): radiculopathy (*n* = 337; 34.6%), polyneuropathy (*n* = 221; 22.7%), and carpal tunnel syndrome (*n* = 218; 22.4%). The presumptive diagnosis was corroborated in 602 (61.8%) patients.

While most referral requests included symptoms (*n* = 774; 79.5%), only 32.1% (*n* = 313) mentioned a physical examination, and regrettably, even fewer met both criteria (*n* = 269; 27.6%). The most frequently reported symptom leading to referral was pain (*n* = 291; 29.9%), followed by paresthesia (*n* = 261; 26.8%) and weakness (*n* = 160; 16.4%). Unfortunately, 199 (20.4%) requests lacked documentation of the symptoms or physical examination that would justify the study. On the other hand, the most frequent electrophysiological diagnoses were normal study results (*n* = 254; 26.1%), carpal tunnel syndrome (*n* = 209; 21.5%), and radiculopathy (*n* = 208; 21.4%).

It was found that only 602 (61.8%) of the clinical diagnoses were corroborated by electrophysiological study; the concordance between the reference clinical diagnosis and the electrophysiological diagnosis was 0.578 (95% CI: 0.537–0.619; *p* < 0.001). Moreover, the concordance between the referral diagnoses and those reported by the EDx laboratory showed that Guillain–Barré syndrome had the highest value (0.826; 95% CI: 0.699–0.952), followed by amyotrophic lateral sclerosis (0.783; 95% CI: 0.613–0.952), myopathy (0.746; 95% CI: 0.639–0.853), and carpal tunnel syndrome (0.751; 95% CI: 0.700–0.802). Diagnoses with lower concordance included multiplex mononeuropathy (0.350; 95% CI: 0.166–0.534) and plexopathy (0.451; 95% CI: 0.255–0.647) (Table 1).

When analyzing concordance by referral service, neurology had the highest concordance (0.853; 95% CI: 0.829–0.878; *p* < 0.001), while neurosurgery had the lowest (0.502; 95% CI: 0.378–0.627; *p* < 0.001) (Table 2).

Finally, we analyzed the relationship between clinical data and the confirmation or rejection of the referral diagnosis, including the most important symptoms or signs (pain, paresthesia, weakness, unspecified in the request, symptoms, and/or signs present together or separately in the request). We found that pain (OR: 3.1; 95% CI: 1.79–5.48; *p* < 0.001) and weakness (OR: 2.46; 95% CI: 1.35–4.48; *p* = 0.003) were significantly associated with a confirmatory diagnosis. Additionally, statistically significant differences were found between the requests sent for pain and paresthesia, with a higher frequency of diagnosis confirmation in those referred for pain and a higher rate of rejection in those referred for paresthesia (Table 3).

## 4. Discussion

In this study, we aimed to determine the concordance between the referral clinical diagnosis and the electrophysiological diagnosis. We compared the diagnostic accuracy of various referral services and assessed the importance of clinical presentation in obtaining concordant electrophysiological results.

It is well known that electrophysiological studies are considered an extension of the clinical evaluation (clinical presentation and physical examination). However, their ability to confirm the clinical diagnosis fluctuates between 40% and 57.6%. This variability can depend on the pathology and the experience of the physician ordering the study [13,14]. Unfortunately, in the present study, approximately 26% of patients undergoing electrodiagnostic evaluation were reported as normal. Although this percentage is lower than that observed in other studies where the frequency of normal study results can reach up to 31.1% to 49.8%, it suggests that adequate documentation of the patient’s clinical presentation and physical examination could significantly improve the diagnostic accuracy of electrophysiological studies and potentially avoid unnecessary testing [15,16,17].

Additionally, it was found that 61.8% of the patients evaluated in the electrodiagnostic laboratory had their referral clinical diagnosis confirmed. This percentage falls within the ranges observed by other authors, where concordance varies between 53.3% and 71.9% [7,18,19]. These variations are likely due to the characteristics of the hospital where the electrodiagnostic studies are conducted. The Hospital General de México ’Dr. Eduardo Liceaga’ has specialists from all areas, with varying levels of experience in neuromusculoskeletal diseases. The requests received by the laboratory are as diverse as the services that submit them, which may result in a lower proportion of concordance between the clinical and electrophysiological diagnoses.

Furthermore, when analyzing the concordance between clinical diagnosis and electrophysiological study, we observed that it reached a concordance coefficient of 0.578 (95% CI: 0.537–0.619; *p* < 0.001). However, this value varied depending on the referring medical specialty. Neurologists had the highest concordance (0.853; 95% CI: 0.829–0.878) and were the service with the most requests during the study period (*n* = 166; 17.4%). On the other hand, neurosurgery, with a total of 124 requests (12.7%) in the same period, showed the lowest concordance (0.502; 95% CI: 0.378–0.627). These variations have been previously reported, demonstrating that the experience of the requesting physician plays a critical role in confirming or rejecting the presumptive diagnosis, especially when the referral comes from specialists not primarily involved in neuromuscular disorders [20].

It was surprising to find such low concordance in the neurosurgery specialty. This finding likely reflects the unnecessary use of electrodiagnostic studies as part of the patient evaluation protocol, rather than as a clinically justified procedure. It is important to remember that diagnostic tests, including electrophysiological studies, should be considered only when they align with the best diagnostic strategy. In neurosurgery, electrodiagnostic studies are primarily used to evaluate peripheral nerve lesions, which may result from compressive mechanisms (e.g., radiculopathy, entrapment syndromes) or trauma (e.g., falls, occupational accidents, penetrating injuries). These lesions may require surgical treatment, with prognosis depending on the degree of injury (neuropraxia, axonotmesis, or neurotmesis). Additionally, electrodiagnostic tools play a crucial role in intraoperative monitoring [21].

The treating physician and the physician responsible for the electrophysiology laboratory must decide whether the study will be useful to confirm the diagnosis, guide subsequent treatment decisions, or assist in the patient’s follow-up and prognosis before subjecting them to potentially uncomfortable or painful procedures unnecessarily [19].

It is important to note that the primary clinical indication justifying the referral of patients for electrodiagnostic studies was pain (*n* = 291; 29.9%), followed by paresthesia (*n* = 261; 26.8%). Nevertheless, the only symptoms and signs associated with a confirmatory diagnosis were pain (OR: 3.1; 95% CI: 1.79–5.48; *p* < 0.001) and weakness (OR: 2.46; 95% CI: 1.35–4.48; *p* = 0.003). It is not surprising that electrodiagnostic studies are used as a valuable diagnostic tool for patients with such signs and symptoms (pain, weakness, paresthesia, areflexia, or cramps) [22]. Nonetheless, it should be considered that electrophysiological studies have limitations in measuring or predicting specific abnormalities of the peripheral nerve and/or muscle and that many of them depend on the operator [23,24].

Despite being considered the gold standard for diagnosing neuromusculoskeletal diseases and having served as the primary diagnostic tool for many neuromuscular conditions, electrodiagnostic studies are often uncomfortable, painful, and difficult to access in most cases. This is why imaging studies have gained relevance in current practice [25]. High-resolution neuromuscular ultrasound has proven to be an instrument that not only confirms electrodiagnostic diagnoses but also helps identify the precise location of lesions when electrodiagnostic studies fall short, thereby enhancing our understanding of the underlying etiology. Initially, its utility was demonstrated in diagnosing local lesions (mononeuropathies) [26,27]. However, its application has expanded to include other peripheral nerve and muscular diseases, demonstrating its value as an essential diagnostic tool. It provides information that can guide treatment decisions, enhance diagnosis, and improve follow-up in patients for whom electrodiagnostic studies may be insufficient, showing good consistency and replicability [28,29].

Moreover, ultrasound is a low-cost, non-invasive, and easily accessible instrument. These characteristics, along with its role as an extension of physical examination, position it as a valuable complement to electrodiagnostic studies, and its combined use should be considered a best practice [30]. Despite its features, versatility, and potential advantages, the use of neuromusculoskeletal ultrasound has not been adopted in most EDx laboratories, and explains why paradigms about the analysis, diagnosis, and follow-up of neuromuscular diseases should be rethought [31].

## 5. Limitations

One of the main limitations of this study is its retrospective nature. Prospective studies are needed to verify whether including a detailed clinical presentation improves clinical and electrophysiological concordance, and also whether to include a neuromuscular ultrasound evaluation. If resources for a prospective study are not available, it is possible to extend the selection period (beyond one year) and even include other types of electrophysiological studies (repetitive nerve stimulation, evoked potentials, blink reflex, etc.) to allow for a comparison between different techniques and to decipher diagnostic consistency in other areas of electrodiagnostic studies.

## 6. Conclusions

The concordance between the results of electrodiagnostic studies and referral requests is limited. Undoubtedly, the experience of the requesting physician in neuromuscular disorders plays a central role in the accuracy and concordance between clinical and electrophysiological findings. Few requests provide sufficient information to guide the electrodiagnostic study, potentially leading to invasive and unnecessary tests, causing patient discomfort, and increasing healthcare costs. Therefore, it is crucial to improve the information contained in the referral and tailor the study to the patient’s clinical presentation and, if it is possible, include imaging studies to improve the diagnosis accuracy.

## 7. Clinical Implications

The findings of this study highlight several critical implications for the appropriate use of electrodiagnostic (EDx) studies in clinical practice. First, the overall concordance rate of 61.8% between referral clinical diagnoses and electrophysiological findings underscores the role of EDx as a complement to the clinical evaluation rather than a standalone diagnostic tool. The variability in diagnostic accuracy across medical specialties, particularly among non-neurology specialists, further emphasizes the importance of a well-documented clinical history and thorough physical examination in optimizing the utility of EDx studies. An inadequate pretest clinical assessment may lead to unnecessary testing and misinterpretation of results, reinforcing the need for clinicians to refine their diagnostic criteria before requesting these studies.

Furthermore, the study confirms that pain and weakness were the primary clinical symptoms associated with a confirmatory EDx diagnosis. This supports the notion that EDx studies are most useful when evaluating patients with neuromuscular symptoms suggestive of nerve dysfunction, while their diagnostic yield diminishes in cases where symptoms are nonspecific or lack clear neurological involvement. Clinicians should therefore prioritize EDx studies for patients presenting with neurological deficits rather than using them indiscriminately in broader patient populations.

Despite being the gold standard for diagnosing neuromuscular disorders, EDx studies have limitations, including procedural discomfort, operator dependency, and limited availability. The increasing role of neuromuscular ultrasound as a complementary tool is particularly relevant, as it can enhance diagnostic accuracy, provide anatomical detail, and improve patient comfort. Given its non-invasive nature, cost-effectiveness, and ability to confirm and localize nerve lesions, its integration into EDx laboratories should be encouraged to improve diagnostic efficiency and patient care.

Overall, this study underscores the necessity of refining the indications for EDx studies, ensuring appropriate patient selection, and integrating complementary diagnostic tools to enhance accuracy and clinical decision making. Establishing standardized referral criteria and promoting interdisciplinary collaboration between referring physicians and neurophysiology specialists will be essential in optimizing the diagnostic yield of EDx studies while minimizing unnecessary procedures.

## Figures and Tables

**Table 1:** Concordance between clinical diagnosis vs. electrophysiology diagnosis: Cohen’s Kappa Coefficient.

Reference Diagnosis	*n* (%)	Cohen’s Kappa	95% CI
Guillain–Barre Syndrome	24 (2.5)	0.826	0.699–0.952
Amyotrophic Lateral Sclerosis	14(1.4)	0.783	0.613–0.952
Carpal Tunnel Syndrome	218 (22.4)	0.751	0.700–0.802
Myopathy	48 (4.9)	0.746	0.639–0.853
Radiculopathy	337 (34.6)	0.619	0.566–0.671
Polyneuropathy	221 (22.7)	0.586	0.522–0.650
Mononeuropathy	64 (6.6)	0.552	0.438–0.666
Plexopathy	24 (2.5)	0.451	0.255–0.647
Multiplex Mononeuropathy	17 (1.7)	0.350	0.166–0.534

**Table 2:** Concordance between clinical diagnosis vs. electrophysiology diagnosis by reference service—Cohen’s Kappa Coefficient.

Reference Service	*n* (%)	Cohen’s Kappa	95% CI
Neurology	169 (17.4)	0.853	0.829–0.878
Rehabilitation	155 (15.9)	0.797	0.775–0.818
Plastic surgery	18 (1.8)	0.758	0.700–0.816
Rheumatology	170 (17.5)	0.742	0.710–0.773
Internal Medicine	33 (3.4)	0.696	0.597–0.795
Orthopedics	250 (25.7)	0.629	0.572–0.685
Pain medicine	29 (3.0)	0.546	0.409–0.682
Neurosurgery	124 (12.7)	0.502	0.378–0.627
Other service	26 (2.7)	0.828	0.781–0.875

**Table 3:** Differences between referral symptoms and signs and electrophysiology diagnosis confirmatory.

Clinical Data	Confirmed Diagnosis *n* (%)	Unconfirmed Diagnosis *n* (%)	*p*
Pain	212 (21.7)	79 (8.1)	<0.001
Weakness	109 (11.2)	51 (5.2)	0.72
Paresthesia	121 (12.4)	140 (14.3)	<0.001
Referral forms with symptoms	470 (48.3)	304 (31.2)	0.171
Referral forms with physical examination	202 (20.7)	111 (11.4)	0.228
Referral forms with symptoms and physical examination	170 (17.5)	99 (10.2)	0.581

## Data Availability

All data generated or analyzed during this study are included in this article. Further inquiries can be directed to the corresponding author. The information was obtained from medical records and is considered confidential and for the sole purpose of this project.

## Abbreviations

The following abbreviations are used in this manuscript:

EDx	Electrodiagnostic
SD	Standard deviation
IQR	Interquartile range
CI	Confidence interval
